# Digital speech biomarkers of cognitive functioning in multilingual Kenyan adults

**DOI:** 10.1002/alz70856_107280

**Published:** 2026-01-10

**Authors:** Adolfo M Garcia, Levi A. Muyela, Alejandro Sosa Welford, Matías Caccia, Nicolás Pelella, Gonzalo Nicolás Pérez, Franco Javier Ferrante, Anne Njoki Gitere, Anne Nyambura Njogu, Erick Muhando, Olivera Nesic‐Taylor, Vaibhav Narayan, Irene Meier, Zul Merali, Chinedu Udeh‐Momoh, Karen Blackmon

**Affiliations:** ^1^ Departamento de Lingüística y Literatura, Facultad de Humanidades, Universidad de Santiago de Chile, Santiago, Chile; ^2^ Global Brain Health Institute (GBHI), University of California San Francisco (UCSF); & Trinity College Dublin, Dublin, Ireland; ^3^ Cognitive Neuroscience Center, University of San Andrés, Victoria, Buenos Aires, Argentina; ^4^ Brain and Mind Institute, Aga Khan University, Nairobi, Kenya; ^5^ Cognitive Neuroscience Centre, CABA, Buenos Aires, Argentina; ^6^ Universidad de Buenos Aires, Buenos Aires, Argentina; ^7^ Consejo Nacional de Investigaciones Científicas y Técnicas (CONICET), Buenos Aires, Argentina; ^8^ Aga Khan University, The Brain and Mind Institute, Nairobi, Kenya; ^9^ Davos Alzheimer's Collaborative, Wayne, PA, USA; ^10^ Wake Forest University, School of Medicine, Winston‐Salem, NC, USA; ^11^ Geisel School of Medicine at Dartmouth, Hanover, NH, USA

## Abstract

**Background:**

Digital speech biomarkers show promise as cost‐effective and scalable diagnostic tools for cognitive decline and dementia in African research and healthcare settings. However, significant gaps remain in language‐specific normative data, cross‐linguistic validation, and standardized protocols for multilingual populations. Targeting cognitively unimpaired multilingual Kenyan adults, here we aim to validate speech timing features from natural language, against performance on standardized culture‐sensitive neuropsychological measures.

**Method:**

This study was funded by Davos Alzheimer's Collaborative and took place in an outpatient clinic at the Aga Khan University in Nairobi, Kenya. Participants completed culturally adapted tests tapping on word list learning, visual memory, naming, cognitive flexibility, and general executive functioning. Using the Toolkit to Examine Lifelike Language (TELL) app, we captured speech timing features (e.g., articulation rate) during standardized tasks (picture description, memory narration, routine recall, video retelling), with no constraints on language‐switching. Upon outlier removal, the sample comprised 49 adults [47% males; mean age: 54 +/‐ 9.21; age range: 44‐77] with broad educational attainment from primary school to doctoral level. Participants spoke at least two languages, with most speaking three (their tribal language, Swahili, and/or English). For each task, machine learning models (ridge, ealastic net, XG‐Boost) were trained with speech‐timing features to predict cognitive test outcomes. The best regressors were selected based on the 95% confidence intervals of R^2^ scores. Pearson's partial correlations between actual and predicted values were computed, controlling for age, sex, education, and language(s) used during the task.

**Results:**

Significant correlations between speech‐predicted and actual cognitive test scores were significant for naming (*r* = .45, *p* = .003), cognitive flexibility (*r* = .37, *p* = .018), and visual memory (*r* = .31, *p* = .046), based on picture description and memory narration. No other correlation was significant.

**Conclusion:**

Digital speech timing features from Kenyan participants are correlated with naming skills and with domain‐general non‐verbal skills vulnerable to aging, irrespective of the language used. These findings underscore the utility of speech biomarkers of cognitive decline for under‐served, multilingual settings. They further represent the first steps towards local normative speech data, supporting the quest for global equity in digital cognitive testing.

